# Comparing Analytical Methods for Gut Microbiome and Aging: Gut Microbiota and Body Weight in the MrOS

**DOI:** 10.1093/geroni/igaa057.3074

**Published:** 2020-12-16

**Authors:** Michelle Shardell, Neeta Parimi, Lisa Langsetmo, Eric Orwoll, James Shikany, Deborah Kado, Peggy Cawthon

**Affiliations:** 1 University of Maryland School of Medicine, Baltimore, Maryland, United States; 2 California Pacific Medical Center, San Francisco, California, United States; 3 University of Minnesota, Minneapolis, Minnesota, United States; 4 Oregon Health & Science University, Portland, Oregon, United States; 5 University of Alabama at Birmingham, Birmingham, Alabama, United States; 6 University of California, San Diego, La Jolla, California, United States

## Abstract

Gut microbiome datasets comprise microbial taxa relative abundances that necessarily sum to 1; analysis ignoring this feature may produce misleading results. We assessed 163 genera from the first batch of Microbiome Ancillary Study (n=530) stool samples and examined associations between microbiota and body weight. We compared conventional Bayesian linear regression (BLR) and network analysis to their compositional counterparts, adjusting for past weight and other covariates. Conventional BLR identified Roseburia and Dialister (positive association) and Coprococcus-1 (negative association) after multiple comparisons adjustment(P<.0125). No conventional network module was associated with weight. Using compositional BLR, men with higher Coprococcus-2 and Acidaminococcus had higher weight, whereas men with higher Coprococcus-1 and Ruminococcus-1 had lower weight (P<.05), but findings were non-significant after multiple comparisons adjustment. Two compositional network modules with respective hub taxa Blautia and Faecalibacterium were associated with weight(P<.01). Findings depended on analytical workflow; compositional analysis is advocated to appropriately handle the sum-to-1 constraint.

